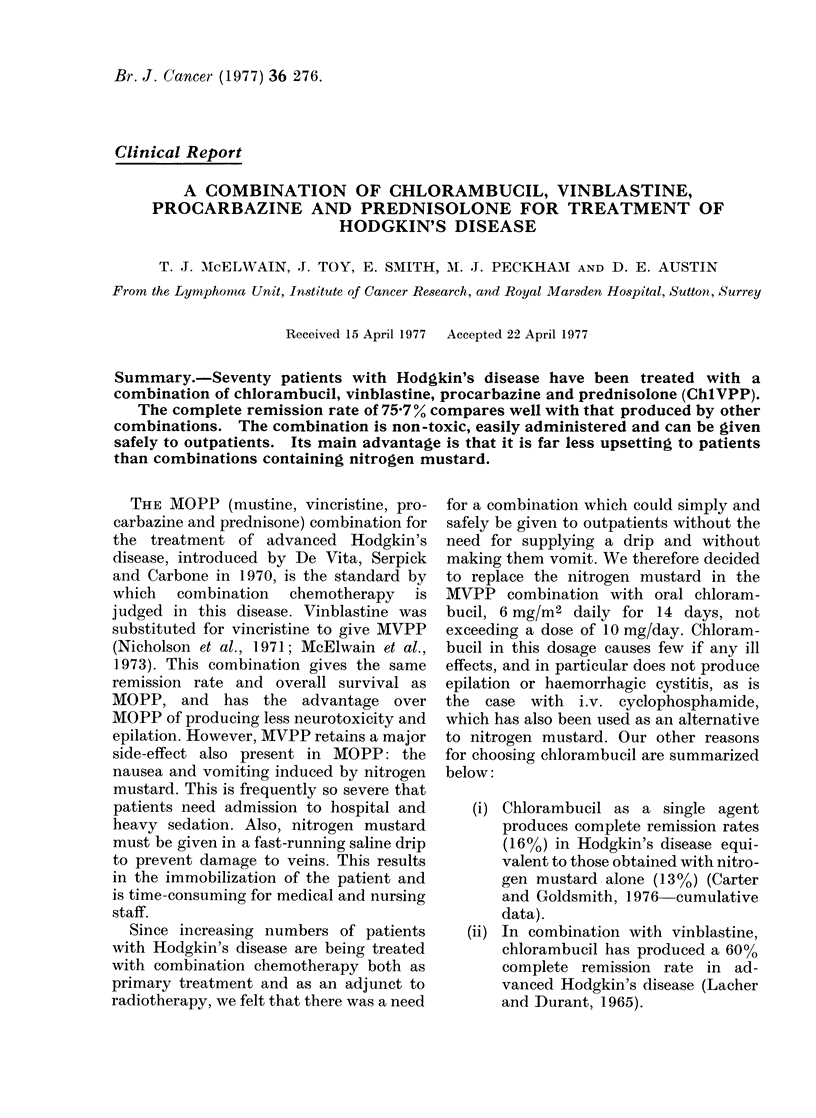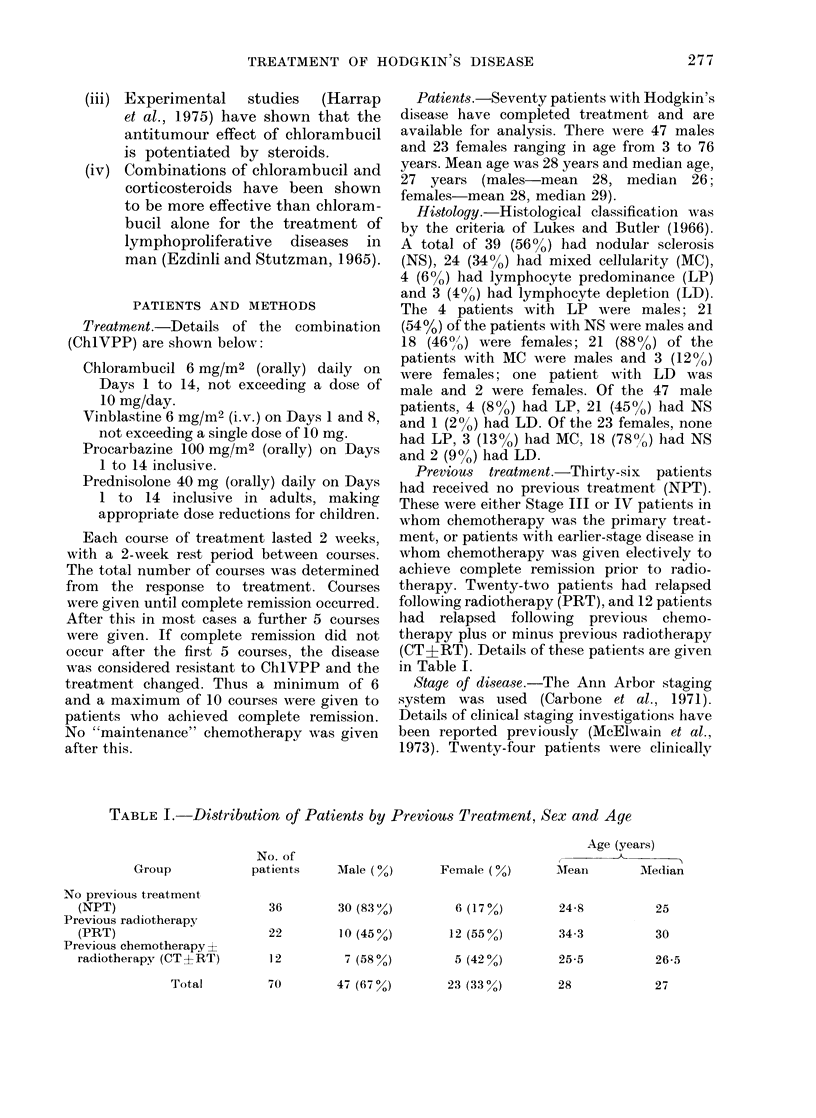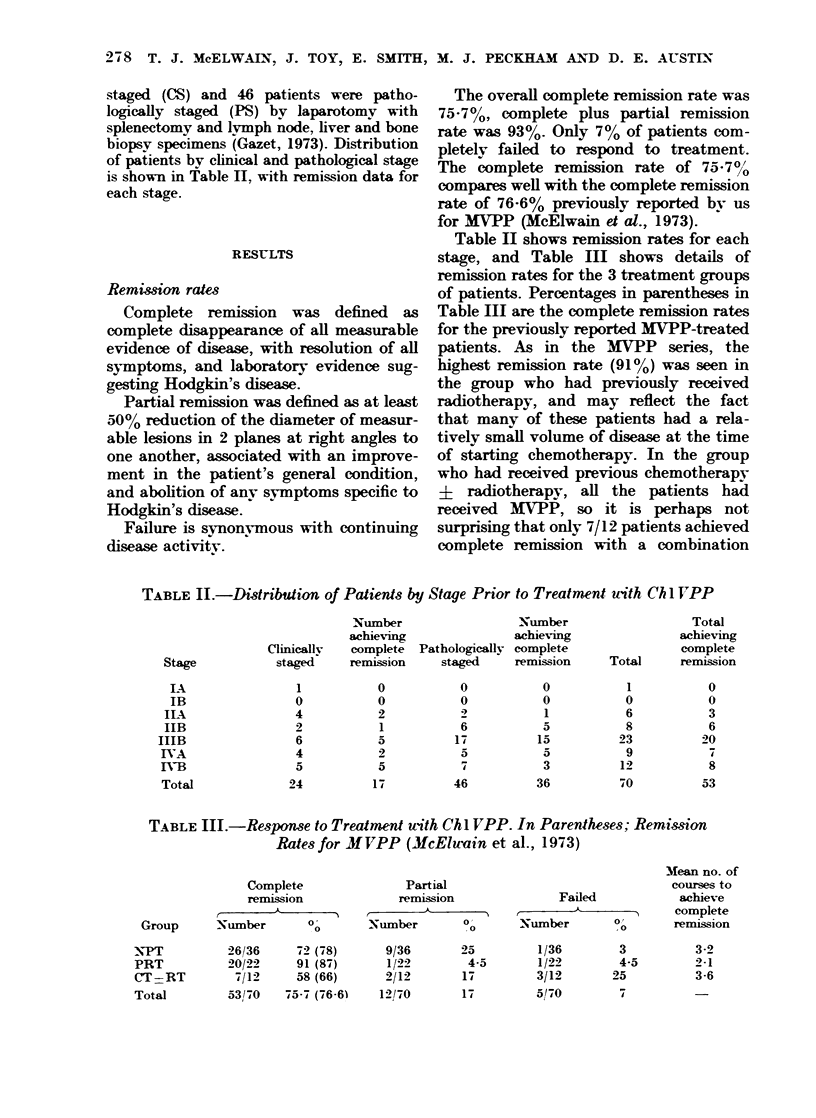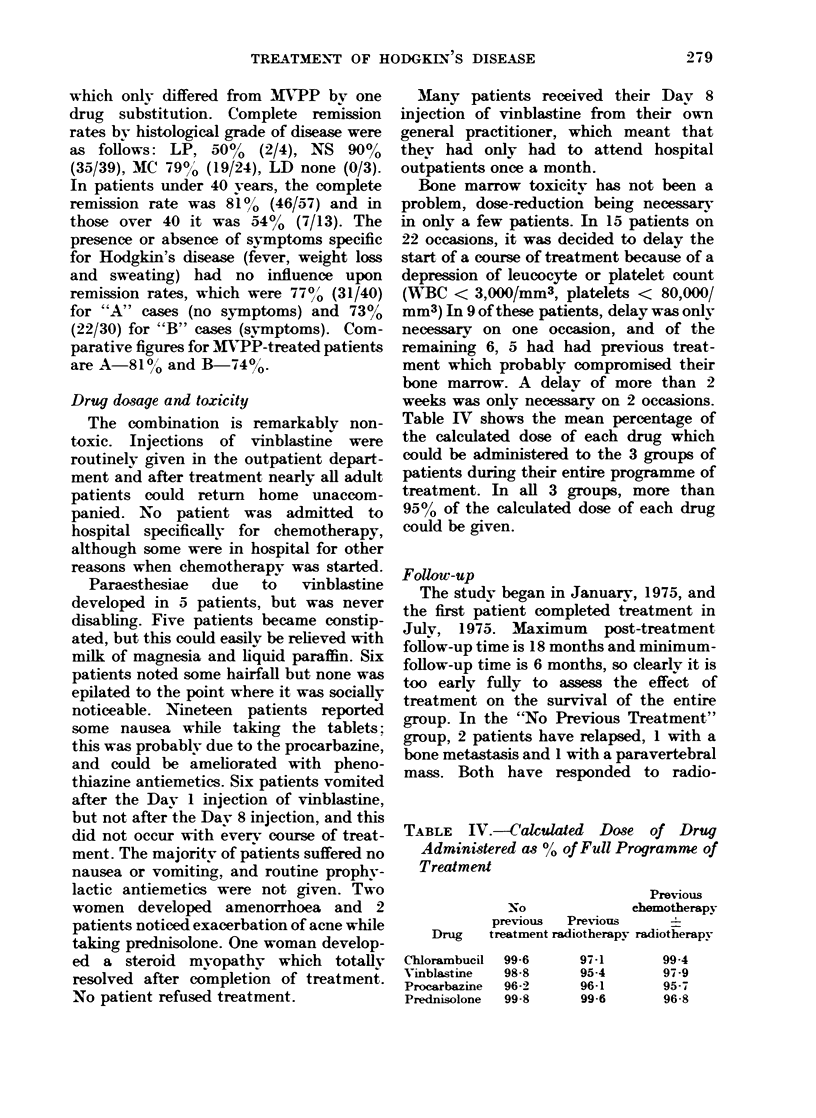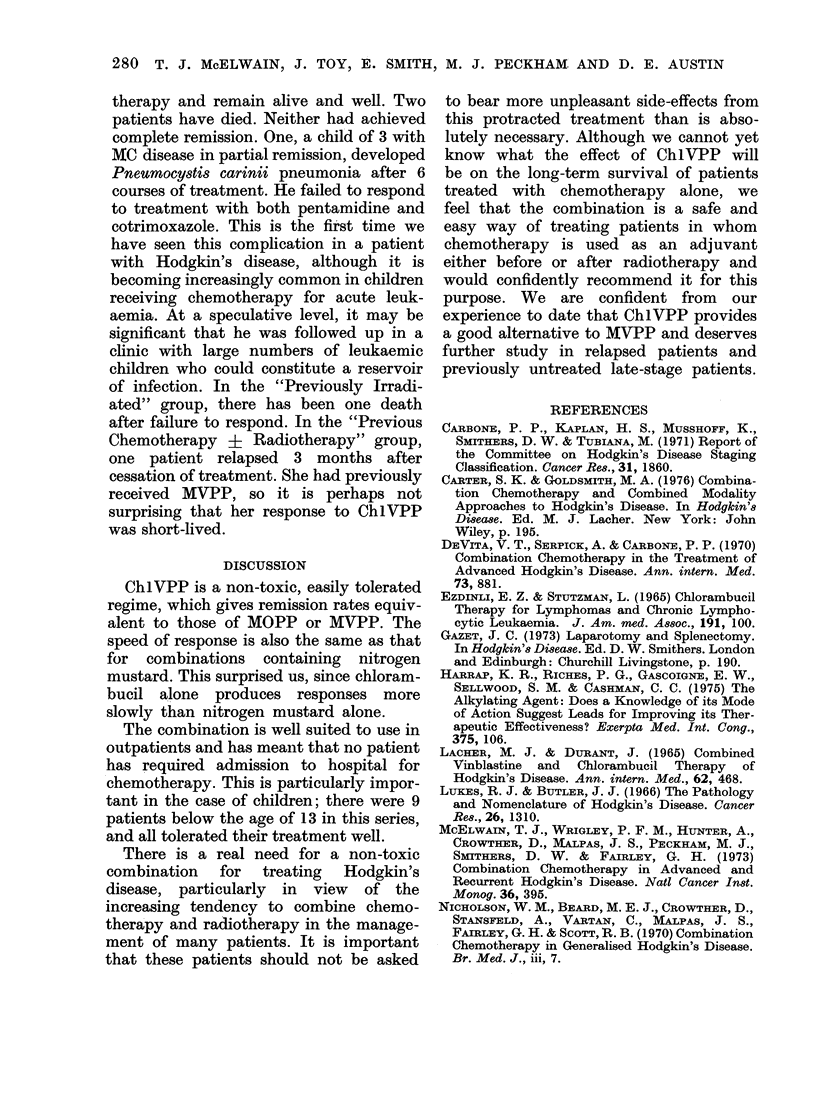# A combination of chlorambucil, vinblastine, procarbazine and prednisolone for treatment of Hodgkin's disease.

**DOI:** 10.1038/bjc.1977.187

**Published:** 1977-08

**Authors:** T. J. McElwain, J. Toy, E. Smith, M. J. Peckham, D. E. Austin

## Abstract

Seventy patients with Hodgkin's disease have been treated with a combination of chlorambucil, vinblastine, procarbazine and prednisolone (Ch1VPP). The complete remission rate of 75-7% compares well with that produced by other combinations. The combination is non-toxic, easily administered and can be given safely to outpatients. Its main advantage is that it is far less upsetting to patients than combinations containing nitrogen mustard.


*Br. J. Cancer* (1977) **36** 276.

### *Clinical Report*

### A COMBINATION OF CHLORAMBUCIL, VINBLASTINE, PROCARBAZINE AND PREDNISOLONE FOR TREATMENT OF HODGKIN'S DISEASE

T. J. McELWAIN, J. TOY, E. SMITH, M. J. PECKHAM AND D. E. AUSTIN

*From the Lymphoma Unit, Institute of Cancer Research, and Royal Marsden Hospital, Sutton, Surrey*

Received 15 April 1977 Accepted 22 April 1977

**Summary.—Seventy patients with Hodgkin's disease have been treated with a combination of chlorambucil, vinblastine, procarbazine and prednisolone (ChlVPP). The complete remission rate of 75·7% compares well with that produced by other combinations. The combination is non-toxic, easily administered and can be given safely to outpatients. Its main advantage is that it is far less upsetting to patients than combinations containing nitrogen mustard.**

THE MOPP (mustine, vincristine, procarbazine and prednisone) combination for the treatment of advanced Hodgkin's disease, introduced by De Vita, Serpick and Carbone in 1970, is the standard by which combination chemotherapy is judged in this disease. Vinblastine was substituted for vincristine to give MVPP (Nicholson *et al.*, 1971; McElwain *et al.*, 1973). This combination gives the same remission rate and overall survival as MOPP, and has the advantage over MOPP of producing less neurotoxicity and epilation. However, MVPP retains a major side-effect also present in MOPP: the nausea and vomiting induced by nitrogen mustard. This is frequently so severe that patients need admission to hospital and heavy sedation. Also, nitrogen mustard must be given in a fast-running saline drip to prevent damage to veins. This results in the immobilization of the patient and is time-consuming for medical and nursing staff.

Since increasing numbers of patients with Hodgkin's disease are being treated with combination chemotherapy both as primary treatment and as an adjunct to radiotherapy, we felt that there was a need for a combination which could simply and safely be given to outpatients without the need for supplying a drip and without making them vomit. We therefore decided to replace the nitrogen mustard in the MVPP combination with oral chlorambucil, 6 mg/m$^2$ daily for 14 days, not exceeding a dose of 10 mg/day. Chlorambucil in this dosage causes few if any ill effects, and in particular does not produce epilation or haemorrhagic cystitis, as is the case with i.v. cyclophosphamide, which has also been used as an alternative to nitrogen mustard. Our other reasons for choosing chlorambucil are summarized below:

(i) Chlorambucil as a single agent produces complete remission rates (16%) in Hodgkin's disease equivalent to those obtained with nitrogen mustard alone (13%) (Carter and Goldsmith, 1976—cumulative data).
(ii) In combination with vinblastine, chlorambucil has produced a 60% complete remission rate in advanced Hodgkin's disease (Lacher and Durant, 1965).

(iii) Experimental studies (Harrap *et al.*, 1975) have shown that the antitumour effect of chlorambucil is potentiated by steroids.
(iv) Combinations of chlorambucil and corticosteroids have been shown to be more effective than chlorambucil alone for the treatment of lymphoproliferative diseases in man (Ezdinli and Stutzman, 1965).

### PATIENTS AND METHODS

*Treatment.*—Details of the combination (ChlVPP) are shown below:

Chlorambucil 6 mg/m$^2$ (orally) daily on Days 1 to 14, not exceeding a dose of 10 mg/day.
Vinblastine 6 mg/m$^2$ (i.v.) on Days 1 and 8, not exceeding a single dose of 10 mg.
Procarbazine 100 mg/m$^2$ (orally) on Days 1 to 14 inclusive.
Prednisolone 40 mg (orally) daily on Days 1 to 14 inclusive in adults, making appropriate dose reductions for children.

Each course of treatment lasted 2 weeks, with a 2-week rest period between courses. The total number of courses was determined from the response to treatment. Courses were given until complete remission occurred. After this in most cases a further 5 courses were given. If complete remission did not occur after the first 5 courses, the disease was considered resistant to ChlVPP and the treatment changed. Thus a minimum of 6 and a maximum of 10 courses were given to patients who achieved complete remission. No "maintenance" chemotherapy was given after this.

*Patients.*—Seventy patients with Hodgkin's disease have completed treatment and are available for analysis. There were 47 males and 23 females ranging in age from 3 to 76 years. Mean age was 28 years and median age, 27 years (males—mean 28, median 26; females—mean 28, median 29).

*Histology.*—Histological classification was by the criteria of Lukes and Butler (1966). A total of 39 (56%) had nodular sclerosis (NS), 24 (34%) had mixed cellularity (MC), 4 (6%) had lymphocyte predominance (LP) and 3 (4%) had lymphocyte depletion (LD). The 4 patients with LP were males; 21 (54%) of the patients with NS were males and 18 (46%) were females; 21 (88%) of the patients with MC were males and 3 (12%) were females; one patient with LD was male and 2 were females. Of the 47 male patients, 4 (8%) had LP, 21 (45%) had NS and 1 (2%) had LD. Of the 23 females, none had LP, 3 (13%) had MC, 18 (78%) had NS and 2 (9%) had LD.

*Previous treatment.*—Thirty-six patients had received no previous treatment (NPT). These were either Stage III or IV patients in whom chemotherapy was the primary treatment, or patients with earlier-stage disease in whom chemotherapy was given electively to achieve complete remission prior to radiotherapy. Twenty-two patients had relapsed following radiotherapy (PRT), and 12 patients had relapsed following previous chemotherapy plus or minus previous radiotherapy (CT±RT). Details of these patients are given in Table I.

*Stage of disease.*—The Ann Arbor staging system was used (Carbone *et al.*, 1971). Details of clinical staging investigations have been reported previously (McElwain *et al.*, 1973). Twenty-four patients were clinically

TABLE I.—*Distribution of Patients by Previous Treatment, Sex and Age*

| Group | No. of patients | Male (%) | Female (%) | Age (years) Mean | Median |
|---|---|---|---|---|---|
| No previous treatment (NPT) | 36 | 30 (83%) | 6 (17%) | 24·8 | 25 |
| Previous radiotherapy (PRT) | 22 | 10 (45%) | 12 (55%) | 34·3 | 30 |
| Previous chemotherapy ± radiotherapy (CT±RT) | 12 | 7 (58%) | 5 (42%) | 25·5 | 26·5 |
| Total | 70 | 47 (67%) | 23 (33%) | 28 | 27 |

staged (CS) and 46 patients were pathologically staged (PS) by laparotomy with splenectomy and lymph node, liver and bone biopsy specimens (Gazet, 1973). Distribution of patients by clinical and pathological stage is shown in Table II, with remission data for each stage.

### RESULTS

*Remission rates*

Complete remission was defined as complete disappearance of all measurable evidence of disease, with resolution of all symptoms, and laboratory evidence suggesting Hodgkin's disease.

Partial remission was defined as at least 50% reduction of the diameter of measurable lesions in 2 planes at right angles to one another, associated with an improvement in the patient's general condition, and abolition of any symptoms specific to Hodgkin's disease.

Failure is synonymous with continuing disease activity.

The overall complete remission rate was 75·7%, complete plus partial remission rate was 93%. Only 7% of patients completely failed to respond to treatment. The complete remission rate of 75·7% compares well with the complete remission rate of 76·6% previously reported by us for MVPP (McElwain *et al.*, 1973).

Table II shows remission rates for each stage, and Table III shows details of remission rates for the 3 treatment groups of patients. Percentages in parentheses in Table III are the complete remission rates for the previously reported MVPP-treated patients. As in the MVPP series, the highest remission rate (91%) was seen in the group who had previously received radiotherapy, and may reflect the fact that many of these patients had a relatively small volume of disease at the time of starting chemotherapy. In the group who had received previous chemotherapy ± radiotherapy, all the patients had received MVPP, so it is perhaps not surprising that only 7/12 patients achieved complete remission with a combination

TABLE II.—*Distribution of Patients by Stage Prior to Treatment with ChlVPP*

| Stage | Clinically staged | Number achieving complete remission | Pathologically staged | Number achieving complete remission | Total | Total achieving complete remission |
|---|---|---|---|---|---|---|
| IA | 1 | 0 | 0 | 0 | 1 | 0 |
| IB | 0 | 0 | 0 | 0 | 0 | 0 |
| IIA | 4 | 2 | 2 | 1 | 6 | 3 |
| IIB | 2 | 1 | 6 | 5 | 8 | 6 |
| IIIB | 6 | 5 | 17 | 15 | 23 | 20 |
| IVA | 4 | 2 | 5 | 5 | 9 | 7 |
| IVB | 5 | 5 | 7 | 3 | 12 | 8 |
| Total | 24 | 17 | 46 | 36 | 70 | 53 |

TABLE III.—*Response to Treatment with ChlVPP. In Parentheses; Remission Rates for MVPP (McElwain* et al., 1973)

| Group | Complete remission | | Partial remission | | Failed | | Mean no. of courses to achieve complete remission |
|---|---|---|---|---|---|---|---|
| | Number | % | Number | % | Number | % | |
| NPT | 26/36 | 72 (78) | 9/36 | 25 | 1/36 | 3 | 3·2 |
| PRT | 20/22 | 91 (87) | 1/22 | 4·5 | 1/22 | 4·5 | 2·1 |
| CT±RT | 7/12 | 58 (66) | 2/12 | 17 | 3/12 | 25 | 3·6 |
| Total | 53/70 | 75·7 (76·6) | 12/70 | 17 | 5/70 | 7 | — |

which only differed from MVPP by one drug substitution. Complete remission rates by histological grade of disease were as follows: LP, 50% (2/4), NS 90% (35/39), MC 79% (19/24), LD none (0/3). In patients under 40 years, the complete remission rate was 81% (46/57) and in those over 40 it was 54% (7/13). The presence or absence of symptoms specific for Hodgkin's disease (fever, weight loss and sweating) had no influence upon remission rates, which were 77% (31/40) for "A" cases (no symptoms) and 73% (22/30) for "B" cases (symptoms). Comparative figures for MVPP-treated patients are A—81% and B—74%.

*Drug dosage and toxicity*

The combination is remarkably non-toxic. Injections of vinblastine were routinely given in the outpatient department and after treatment nearly all adult patients could return home unaccompanied. No patient was admitted to hospital specifically for chemotherapy, although some were in hospital for other reasons when chemotherapy was started.

Paraesthesiae due to vinblastine developed in 5 patients, but was never disabling. Five patients became constipated, but this could easily be relieved with milk of magnesia and liquid paraffin. Six patients noted some hairfall but none was epilated to the point where it was socially noticeable. Nineteen patients reported some nausea while taking the tablets; this was probably due to the procarbazine, and could be ameliorated with phenothiazine antiemetics. Six patients vomited after the Day 1 injection of vinblastine, but not after the Day 8 injection, and this did not occur with every course of treatment. The majority of patients suffered no nausea or vomiting, and routine prophylactic antiemetics were not given. Two women developed amenorrhoea and 2 patients noticed exacerbation of acne while taking prednisolone. One woman developed a steroid myopathy which totally resolved after completion of treatment. No patient refused treatment.

Many patients received their Day 8 injection of vinblastine from their own general practitioner, which meant that they had only had to attend hospital outpatients once a month.

Bone marrow toxicity has not been a problem, dose-reduction being necessary in only a few patients. In 15 patients on 22 occasions, it was decided to delay the start of a course of treatment because of a depression of leucocyte or platelet count (WBC $< 3,000/mm^3$, platelets $< 80,000/mm^3$) In 9 of these patients, delay was only necessary on one occasion, and of the remaining 6, 5 had had previous treatment which probably compromised their bone marrow. A delay of more than 2 weeks was only necessary on 2 occasions. Table IV shows the mean percentage of the calculated dose of each drug which could be administered to the 3 groups of patients during their entire programme of treatment. In all 3 groups, more than 95% of the calculated dose of each drug could be given.

*Follow-up*

The study began in January, 1975, and the first patient completed treatment in July, 1975. Maximum post-treatment follow-up time is 18 months and minimum-follow-up time is 6 months, so clearly it is too early fully to assess the effect of treatment on the survival of the entire group. In the "No Previous Treatment" group, 2 patients have relapsed, 1 with a bone metastasis and 1 with a paravertebral mass. Both have responded to radio-

TABLE IV.—*Calculated Dose of Drug Administered as % of Full Programme of Treatment*

| Drug | No previous treatment | Previous radiotherapy | Previous chemotherapy ± radiotherapy |
|---|---|---|---|
| Chlorambucil | 99·6 | 97·1 | 99·4 |
| Vinblastine | 98·8 | 95·4 | 97·9 |
| Procarbazine | 96·2 | 96·1 | 95·7 |
| Prednisolone | 99·8 | 99·6 | 96·8 |

therapy and remain alive and well. Two patients have died. Neither had achieved complete remission. One, a child of 3 with MC disease in partial remission, developed *Pneumocystis carinii* pneumonia after 6 courses of treatment. He failed to respond to treatment with both pentamidine and cotrimoxazole. This is the first time we have seen this complication in a patient with Hodgkin's disease, although it is becoming increasingly common in children receiving chemotherapy for acute leukaemia. At a speculative level, it may be significant that he was followed up in a clinic with large numbers of leukaemic children who could constitute a reservoir of infection. In the "Previously Irradiated" group, there has been one death after failure to respond. In the "Previous Chemotherapy $\pm$ Radiotherapy" group, one patient relapsed 3 months after cessation of treatment. She had previously received MVPP, so it is perhaps not surprising that her response to ChlVPP was short-lived.

### DISCUSSION

ChlVPP is a non-toxic, easily tolerated regime, which gives remission rates equivalent to those of MOPP or MVPP. The speed of response is also the same as that for combinations containing nitrogen mustard. This surprised us, since chlorambucil alone produces responses more slowly than nitrogen mustard alone.

The combination is well suited to use in outpatients and has meant that no patient has required admission to hospital for chemotherapy. This is particularly important in the case of children; there were 9 patients below the age of 13 in this series, and all tolerated their treatment well.

There is a real need for a non-toxic combination for treating Hodgkin's disease, particularly in view of the increasing tendency to combine chemotherapy and radiotherapy in the management of many patients. It is important that these patients should not be asked to bear more unpleasant side-effects from this protracted treatment than is absolutely necessary. Although we cannot yet know what the effect of ChlVPP will be on the long-term survival of patients treated with chemotherapy alone, we feel that the combination is a safe and easy way of treating patients in whom chemotherapy is used as an adjuvant either before or after radiotherapy and would confidently recommend it for this purpose. We are confident from our experience to date that ChlVPP provides a good alternative to MVPP and deserves further study in relapsed patients and previously untreated late-stage patients.

### REFERENCES

Carbone, P. P., Kaplan, H. S., Musshoff, K., Smithers, D. W. & Tubiana, M. (1971) Report of the Committee on Hodgkin's Disease Staging Classification. *Cancer Res.*, **31**, 1860.

Carter, S. K. & Goldsmith, M. A. (1976) Combination Chemotherapy and Combined Modality Approaches to Hodgkin's Disease. In *Hodgkin's Disease*. Ed. M. J. Lacher. New York: John Wiley, p. 195.

DeVita, V. T., Serpick, A. & Carbone, P. P. (1970) Combination Chemotherapy in the Treatment of Advanced Hodgkin's Disease. *Ann. intern. Med.* **73**, 881.

Ezdinli, E. Z. & Stutzman, L. (1965) Chlorambucil Therapy for Lymphomas and Chronic Lymphocytic Leukaemia. *J. Am. med. Assoc.*, **191**, 100.

Gazet, J. C. (1973) Laparotomy and Splenectomy. In *Hodgkin's Disease*. Ed. D. W. Smithers. London and Edinburgh: Churchill Livingstone, p. 190.

Harrap, K. R., Riches, P. G., Gascoigne, E. W., Sellwood, S. M. & Cashman, C. C. (1975) The Alkylating Agent: Does a Knowledge of its Mode of Action Suggest Leads for Improving its Therapeutic Effectiveness? *Exerpta Med. Int. Cong.*, **375**, 106.

Lacher, M. J. & Durant, J. (1965) Combined Vinblastine and Chlorambucil Therapy of Hodgkin's Disease. *Ann. intern. Med.*, **62**, 468.

Lukes, R. J. & Butler, J. J. (1966) The Pathology and Nomenclature of Hodgkin's Disease. *Cancer Res.*, **26**, 1310.

McElwain, T. J., Wrigley, P. F. M., Hunter, A., Crowther, D., Malpas, J. S., Peckham, M. J., Smithers, D. W. & Fairley, G. H. (1973) Combination Chemotherapy in Advanced and Recurrent Hodgkin's Disease. *Natl Cancer Inst. Monog.* **36**, 395.

Nicholson, W. M., Beard, M. E. J., Crowther, D., Stansfeld, A., Vartan, C., Malpas, J. S., Fairley, G. H. & Scott, R. B. (1970) Combination Chemotherapy in Generalised Hodgkin's Disease. *Br. Med. J.*, iii, 7.